# Supplementary material for: Neurodevelopment of 24 children born in Brazil with congenital Zika syndrome in 2015: a case series study
Source: BMJ Open. 2018 Jul 16;8(7):e021304. doi: 10.1136/bmjopen-2017-021304 (PMC6082469; doi:10.1136/bmjopen-2017-021304)
Supplement: Author's manuscript [file bmjopen-2017-021304.draft_revisions.pdf]

BMJ Open is committed to open peer review. As part of this commitment we make the peer review history of every article we publish publicly available.

When an article is published we post the peer reviewers' comments and the authors' responses online. We also post the versions of the paper that were used during peer review. These are the versions that the peer review comments apply to.

The versions of the paper that follow are the versions that were submitted during the peer review process. They are not the versions of record or the final published versions. They should not be cited or distributed as the published version of this manuscript.

BMJ Open is an open access journal and the full, final, typeset and author-corrected version of record of the manuscript is available on our site with no access controls, subscription charges or pay-per-view fees (http://bmjopen.bmj.com).

If you have any questions on BMJ Open's open peer review process please email info.bmjopen@bmj.com

NEURODEVELOPMENT OF 24 CHILDREN WITH CONGENITAL ZIKA SYNDROME – CASE SERIES STUDY

Journal:	*BMJ Open*
Manuscript ID	bmjopen-2017-021304
Article Type:	Research
Date Submitted by the Author:	22-Dec-2017
Complete List of Authors:	Alves, Lucas; Instituto de Medicina Integral Professor Fernando Figueira, Paediatric Neurology<br>Paredes, Camila; Faculdade Pernambucana de Saude, Teaching Department<br>Silva, Germanna; Faculdade Pernambucana de Saude<br>Mello, Julia; Instituto de Medicina Integral Professor Fernando Figueira, Department of Paediatrics<br>Alves, João; IMIP, Department of Paediatrics
Keywords:	Developmental neurology & neurodisability < PAEDIATRICS, Paediatric infectious disease & immunisation < PAEDIATRICS, Paediatric neurology < PAEDIATRICS

SCHOLARONE™
Manuscripts

NEURODEVELOPMENT OF 24 CHILDREN WITH CONGENITAL ZIKA SYNDROME – CASE SERIES STUDY

Lucas V Alves, Camila E Paredes, Germanna C Silva, Júlia G Mello, João G Alves

Department of Paediatrics Neurology, Instituto de Medicina Integral Prof. Fernando Figueira (IMIP), Rua dos Coelhos, 300, Boa Vista, Recife, Brazil, Postcode 50070-550 Lucas V Alves

Pediatric Neurologist

Teaching Department, Faculdade Pernambucana de Saúde (FPS) Rua Jean Émile Favre, 422, Imbiribeira, Recife, Brazil, Postcode 51200-060 Camila E Paredes

Medical Student

Teaching Department, Faculdade Pernambucana de Saúde (FPS), Rua Jean Émile Favre, 422, Imbiribeira, Recife, Brazil, Postcode 51200-060. Germanna V Silva

Medical Student

Department of Paediatrics, Instituto de Medicina Integral Prof. Fernando Figueira (IMIP), Rua dos Coelhos, 300, Boa Vista, Recife, Brazil, Postcode 500070-550 Júlia G Mello,

Pediatrician

Department of Pediatrics, Instituto de Medicina Integral Prof. Fernando Figueira (IMIP), Rua dos Coelhos, 300, Boa Vista, Recife, Brazil, Postcode 500070-550.

João G Alves

Pediatrician

Correspondence to:

João Guilherme Alves

For peer review only

Instituto de Medicina Integral Professor Fernando Figueira (IMIP)

Rua dos Coelhos, 300 – Boa Vista, Recife – PE. Brazil.

Postcode 500070-550.

Email: joaoguilherme@imip.org.br

Phone: +55 (81) 999746351

ABSTRACT

Objective: To describe the neurodevelopment of children with congenital Zika syndrome during the second year of life.

Design: Case series study.

Setting: Instituto de Medicina Integral Prof. Fernando Figueira (IMIP), Pernambuco state, Brazil.

Participants: 24 children with congenital Zika syndrome born during the microcephaly outbreak in Brazil, 2015 and accompained at IMIP during their second year of life.

Main outcome measure: Denver development test II, head circumference and clinical neurological examination.

Results: All children presented neurodevelopment delay: for an average chronological age of 19.9 months the equivalent age for language was 2.1 months, gross motor 2.7 months, fine motor/adaptative 3.1 months and personal/social 3.4 months. Head circumference maintained below the 3rd percentile for age and gender; growth rate up to the second year of life was 10.3cm (expected growth 13cm). Muscle tone was increased in 23/24 (95.5%) of children, musculo-tendinous reflexes were increased in the whole sample and clonus was present in 18/24 (77.3%). All except one had epilepsy.

For peer review only - http://bmjopen.bmj.com/site/about/guidelines.xhtml

Conclusion: Children born with microcephaly associated with congenital Zika vírus have a significant delay in neurodevelopment.

Key-words: Zika vírus, congenital Zika syndrome, neurodevelopment

Strengths and limitations of this study

For the first time in children with congenital Zika syndrome:

- The neurodevelopment during the first two years of life is reported
- Denver development screening test II was applied
- Head circumference was measured from birth to around two years old

Introduction

After the outbreak of congenital Zika syndrome in Brazil (2015/2016), healthcare professionals are faced with a population of children with microcephalic and many neurological manifestations (1,2). These children may present epilepsy, abnormalities of tone or movement, including marked hypertonia and signs of extrapyramidal involvement, congenital limb contractures, dysphagia, sensorineural hearing loss and visual involvement (3,4). However there are no published follow-up studies reporting the neurodevelopment, head growth and evolution of neurologic manifestations in children with congenital Zika syndrome. Some of these children have been followed at IMIP and they are now around two years old. Our aim is to describe the neurodevelopment,

For peer review only

head growth and neurologic clinical manifestations of these children with congenital Zika syndrome during the first two years of life.

Methods

A case series study was conducted at IMIP, Recife, Brazil, between January and August 2017. At the time of the study 24 children with microcephaly and congenital Zika syndrome were being followed at the Department of Paediatric Neurology of IMIP and were close to completing the second year of life. All this children were born with microcephaly and congenital Zika syndrome, as defined by the WHO (https://www.cdc.gov/pregnancy/zika/research/microcephaly-case-definitions.html5). In the age between 18 and 24 months they were submitted to a complete neurological evaluation including the application of the Denver development test II. This study was approved by IMIP Research Ethical Committee (CAAE 6167876.1.0000.5201) and an informed consent was signed by the parents of the studied children.

All children were born with a head circumference below the 3rd percentile for gestational age and gender. Congenital Zika syndrome was characterized by microcephaly associated with the following neuroimages pattern: cerebral calcifications, ventriculomegaly, malformation of cortical development, hypoplasia of the cerebellum or brainstem, and abnormalities of white matter; laboratory findings excluded STORCH (syphilis, toxoplasmosis, rubella, cytomegalovirus, and herpesvirus) infections in the mother or baby, or both; and a serum or cerebrospinal fluid sample positive for IgM anti-Zika.

A clinical evaluation included a complete physical and neurological examination, measurement of head circumference and psychomotor development assessment by checking if the chronological acquisition of the main neurological milestones were appropriated for age. The Denver development screening test II was also applied. This test is a

For peer review only

screening tool for child development that evaluates children ages 0-6 years old. Denver II includes 125 itens in four sections: psico-social (aspects regarding the socialization of the child inside and outside the family environment), language (production of sound, ability to recognize, understand and use the language), gross motor (body motor control, how to sit and walk) and fine motor adaptive (eye-hand coordination, manipulation of small objects). The possible outcomes were classified as normal, delay and non-testable. Clinical exam and Denver development screening test II were applied by a neuropediatrician specialist.

Results

24 children with congenital Zika syndrome were evaluated, being 14 (58.3%) female; age ranged from 16 to 24 months (19.7 m). Mothers age ranged from 15 to 39 years and 10/24 (41.6%) were primiparus; 17/24 had a microcephaly antenatal diagnostic by fetal ultrasonographic exam.

Two children were preterm (31 and 35 gestational week). Head circumference growth is shown at table 1; mean growth of head perimeter from birth to the age of clinical evaluation was 10.3 cm. 8/24 (33.3%) of the children had hospital admissions: non-controlled epilepsy (2), ventriculoperitoneal shunt (2) and infection (3) – diarrheia, urinary tract infection and pneumonia. Almost all children (23/24) had recurrent episodes of convulsion.

Neurologic evaluation showed that all children presented impairment of neuropsychomotor development; none stood with support, walked or say a word. Denver screening test II results according to the equivalente age are shown at table 2; greater impairment was observed in language and lower impairment in psico-social section. The mean age of the clinical

For peer review only

examination was 19.9 months and equivalente age for language, gross motor, fine motor/adaptative and personal/social were respectively: 2.1 months, 2.7 months, 3.1 months and 3.4 months.

Tone was increased in 23/24 (95.5%) of children, musculo-tendinous reflexes were increased in the whole sample and clonus was present in 18/24 (77.3%).

Discussion

A severe impairment of theneuropsychomotor development of children with congenital Zika syndrome was observed. All children at about 2 years of age could not stand alone, walk or say a word. It should be noted that these children were accompanied in a teaching hospital and were assisted by several health professionals including physicians, physiotherapists, speech therapists, occupational therapists, psychologists and nurses. This seems to assume that the prognosis of these children is very serious even with all the therapeutic accompaniment.

Congenital microcephaly regardless of cause presents a significant risk for delay across all aspects of development and for long term disability. Serious development delay was found in all 24 children with congenital Zika síndrome around 2 years of age. Gordon-Lipkine et al (5) studying children with congenital microcephaly of diferente etiologies found that 16/22 (73%) had delay in development: gross motor (65%), fine-motor (59%) and language (59%).

Head growth during the first months of life remained below the normal values (observed 10.3 cm and expected 13/14cm) and all children had a head circumference below the 3rd percentile for age and gender. Brain size correlates with head circumference. Decreased growth velocity of the head may indicate that there is a disruption in neurodevelopment (6).

For peer review only

Zika vírus can infect and obliterate cells from the central nervous system, such as progenitors, neurons, and glial cells. During fetal life central nervous system presente mainly a progressive CNS lesions and slowing rate of growth of the fetal head (7). Neural progenitor cells infections leads to less cell migration, neurogenesis impairment, cell death and,

consequently, microcephaly in newborns. The downsizing of the brain can be directly associated with defective development of the cortical layer (7). All this seems to cause damage for the rest of life.

Almost all studied children has epilepsy. Some studies have shown that convulsive epilepsy worsens the development delay of children with microcephaly (5, .

The Denver development test II is culturally adapted to Brazil (8). The Denver test II was applied in children with ages between 17 and 24 months and results obtained were equivalent to the development for the age of 1 to 8 months. This means that a serious impact on the development is associated with congenital Zika syndrome.

Our study has strength and limitations. For the first time the neurodevelopment of infants born with microcephaly associated with Zika virus is reported. Unfortunately, due to operational difficulties we were unable to study all 57 children followed at IMIP. The Denver development test II was applied by only one evaluator but the delays in the marks of neurodevelopment were very clear.

Conclusion

Children born with microcephaly associated with congenital Zika vírus have a significant delay in neurodevelopment during their second year of life. As this study was the first to describe the neurodevelopment of children with congenital Zika syndrome others follow-up studies are needing to confirm

For peer review only

these findings. We urgently need to optimize the provision of healthcare and improve the quality of life of these patients.

Contributors

LVA and JGM contributed to conception of the work; LVA, CEP and GCS, contributed to the acquisition of data; LVA, JGM, JGA contributed to the analysis and interpretation of data for the work; and LVA is the guarantor.

LVA, CEP, GCS, JGM and JGA drafted the work and revised it critically. LVA, CEP, GCS, JGM and JGA approved the version to be published.

Funding

No external funding provided.

Competing interests

All authors have completed the ICMJE uniform disclosure form at www.icmje.org/coi_disclosure.pdf and declare: no support from any organisation for the submitted work; no financial relationships with any organisations that might have an interest in the submitted work in the previous three years; no other relationships or activities that could appear to have influenced the submitted work.

Ethical approval

This study was approved by the IMIP ethics committee and the children's carers gave informed consent for the publication of the results.

Data sharing

Clinical data is available from the corresponding author at joaoguilherme@imip.org.br

Transparency

The lead author (LVA) affirms that the manuscript is an honest, accurate, and transparent account of the study being reported; that no important aspects of the study have been omitted; and that any discrepancies from the study as planned have been explained.

References

1 - Passi D, Sharma S, Dutta SR, Ahmed M. Zika Virus Diseases - The New Face of an Ancient Enemy as Global Public Health Emergency (2016): Brief Review and Recent Updates. Int J Prev Med. 2017;8:6. doi: 10.4103/2008-7802.199641.

2 – Triunfo M. Microcephaly in Brazil: confidence builds in Zika connection. Lancet Infect Dis. 2016;16(5):527-528. doi: 10.1016/S1473-3099(16)30015-9

3 - Moore CA, Staples JE, Dobyns WB, et al. Characterizing the pattern of anomalies in congenital Zika syndrome for pediatric clinicians. JAMA Pediatr 2017;171:288– PubMed ;95.

4 - Leal MC, Muniz LF, Ferreira TS, et al. Hearing loss in infants with microcephaly and evidence of congenital Zika virus infection—Brazil, November 2015–May 2016. MMWR Morb Mortal Wkly Rep 2016;65:917–9.*14*,*15*.

5 - Gordon-Lipkine E, Gentner MB, German R, Leppert ML. Neurodevelopmental outcomes in 22 children with microcephaly of different etiologies. J Child Neurol 2017;32(9):804 PubMed -9.

6 - Saad T, PennaeCosta AA, de Góes FV, de Freitas M, de Almeida JV, de SantaIgnêz LJ, Amancio AP, Alvim RJ, Antunes Kramberger LA. Neurological manifestations of congenital Zika virus infection. Childs Nerv Syst. 2017 Nov 10. doi: 10.1007/s00381-017-3634-4.

7 - Sarno R, Aquino M, Pimentel K, Cabral R, Costa G, Bastos F, Brites C. Progressive lesions of central nervous system in microcephalic fetuses with suspected congenital Zika vírus syndrome. Ultrasound Obstet Gynecol 2016. Doi: 10.1002/uog.17303.

8 - Russo FB, Jungmann P, Beltrão-Braga PCB Zika infection and the development of neurological defects. Cell Microbiol. 2017 Jun;19(6). doi: 10.1111/cmi.12744.

9 - Pinto FCA, Isotani SM, Sabatés AL, Perissinoto J. Denver II: proposed behaviors compared to those of children from São Paulo. Revista CEFAC. 2015. Vol 17(4). p.1262-1269

For peer review only

Tabel 1– Head circumference growth from birth to current age

Gender	Current age (months)	Head circumference at birth (cm)	Current head circumference (cm)	Head circumferenc expected (cm)
F	19	31	41	45.7
F	18	30	38	45.6
M	21	26	36	46.4
M	20	27	39	46.3
F	21	32	41	45.8
M	22	23	37	47.5
F	20	30	37	45.8
M	19	31	44	47.2
M	17	27	35	47.0
F	20	29	43.5	45.8
F	21	28	36	45.9
M	21	29	41	46.4
F	19	26	37.5	45.7
M	19	29	45	47.2
F	19	28	37	45.7
F	19	29.5	39	45.7
M	18	27	42	47.2
F	19	31.5	42	45.7
F	22	25	35.5	47.0
F	22	31	40	47.0
M	24	29	37.5	47.7
F	19	30.5	42.5	45.7

For peer review only

M	16	30	41	45.6
F	24	31	43	47.0

For peer review only

Tabel 2– Denver Development Screening Test II in 24 children with congenital Zika syndrome

Current age (months)	Language (equivalence in months)	Gross motor (equivalence in months)	Fine motor/adaptative (equivalence in months)	Person (equiva mon
19	1	1	1	
18	1	4	4	
21	1	4	4	
20	1	1	1	
21	4	4	4	
22	1	1	1	
20	1	1	1	
19	6	7	7	
17	1	1	1	
20	7	7	7	
21	1	1	1	
21	1	4	8	
19	1	1	4	
19	1	1	1	
19	4	5	4	
19	4	1	1	
18	1	1	1	
19	1	1	1	
22	4	4	6	
22	3	4	4	

For peer review only

24	1	4	4
19	1	3	3
16	2	2	3
24	2	3	3

For peer review only

CHECKLIST

STROBE Statement —checklist of items that should be included in reports of observational studies

(1) Title and abstract: (a) Indicate the study's design with a commonly used term in the title or the abstract **OK** (b) Provide in the abstract an informative and balanced summary of what was done and what was found Introduction **OK**
(2) Background/rationale: Explain the scientific background and rationale for the investigation being reported **OK**
(3) Objectives: State specific objectives, including any prespecified hypotheses **OK**
(4) Methods Study design Present key elements of study design early in the paper **OK**
(5) Setting Describe the setting, locations, and relevant dates, including periods of recruitment, exposure, follow-up, and data collection **OK**
(6) Variables Clearly define all outcomes, exposures, predictors, potential confounders, and effect modifiers. Give diagnostic criteria, if applicable **OK**
(7) Data sources/ measurement For each variable of interest, give sources of data and details of methods of assessment (measurement). Describe comparability of assessment methods if there is more than one group **OK**
(8) Bias Describe any efforts to address potential sources of bias **OK**
(9) Study size Explain how the study size was arrived – **Not applicable**
(10) Quantitative variables Explain how quantitative variables were handled in the analyses. **– Not applicable**
(11) Results (a) Report numbers of individuals at each stage of study—eg numbers potentially eligible, examined for eligibility, confirmed eligible, included in the study, completing follow-up, and analysed (b) Give reasons for non-participation at each stage Participants 13* (c) Consider use of a flow diagram (a) Give characteristics of study participants (eg demographic, clinical, social) and information on exposures and potential confounders (b) Indicate number of participants with missing data for each variable of interest - **OK**
(12) Descriptive data – OK
(13) Outcome data Cross-sectional study—Report numbers of outcome events or summary measures – **OK**
(14) Main results If relevant, consider translating estimates of relative risk into absolute risk for a meaningful time period – **Not applicable**
(15) Other analyses Report other analyses done—eg analyses of subgroups and interactions, and sensitivity – **Not applicable**
(16) analyses Discussion Key results Summarise key results with reference to study objectives Limitations - **OK**
(17) Discuss limitations of the study, taking into account sources of potential bias or imprecision. Discuss both direction and magnitude of any potential bias Interpretation – **OK**
(18) Give a cautious overall interpretation of results considering objectives, limitations, multiplicity of analyses, results from similar studies, and other relevant evidence – **OK**
(19) Generalisability Discuss the generalisability (external validity) of the study results Other information Funding - **OK**

For peer review only

(20) Give the source of funding and the role of the funders for the present study and, if applicable, for the original study on which the present article is based – **Not applicable**

For peer review only

BMJ Open

NEURODEVELOPMENT OF 24 CHILDREN BORN IN BRAZIL WITH CONGENITAL ZIKA SYNDROME IN 2015 – CASE SERIES STUDY

Journal:	*BMJ Open*
Manuscript ID	bmjopen-2017-021304.R1
Article Type:	Research
Date Submitted by the Author:	12-Apr-2018
Complete List of Authors:	Alves, Lucas; Instituto de Medicina Integral Professor Fernando Figueira, Paediatric Neurology<br>Paredes, Camila; Faculdade Pernambucana de Saude, Teaching Department<br>Silva, Germanna; Faculdade Pernambucana de Saude<br>Mello, Julia; Instituto de Medicina Integral Professor Fernando Figueira, Department of Paediatrics<br>Alves, João; IMIP, Department of Paediatrics
**Primary Subject Heading**:	Infectious diseases
Secondary Subject Heading:	Neurology, Paediatrics
Keywords:	Developmental neurology & neurodisability < PAEDIATRICS, Paediatric infectious disease & immunisation < PAEDIATRICS, Paediatric neurology < PAEDIATRICS

SCHOLARONE™
Manuscripts

NEURODEVELOPMENT OF 24 CHILDREN BORN IN BRAZIL WITH CONGENITAL ZIKA SYNDROME IN 2015 – CASE SERIES STUDY

Lucas V Alves, Camila E Paredes, Germanna V Silva, Júlia G Mello, João G Alves

Department of Paediatrics Neurology, Instituto de Medicina Integral Prof. Fernando Figueira (IMIP), Rua dos Coelhos, 300, Boa Vista, Recife, Brazil, Postcode 50070-550 Lucas V Alves

Pediatric Neurologist

Teaching Department, Faculdade Pernambucana de Saúde (FPS) Rua Jean Émile Favre, 422, Imbiribeira, Recife, Brazil, Postcode 51200-060 Camila E Paredes

Medical Student

Teaching Department, Faculdade Pernambucana de Saúde (FPS), Rua Jean Émile Favre, 422, Imbiribeira, Recife, Brazil, Postcode 51200-060. Germanna V Silva

Medical Student

Department of Paediatrics, Instituto de Medicina Integral Prof. Fernando Figueira (IMIP), Rua dos Coelhos, 300, Boa Vista, Recife, Brazil, Postcode 500070-550 Júlia G Mello,

Pediatrician

Department of Pediatrics, Instituto de Medicina Integral Prof. Fernando Figueira (IMIP), Rua dos Coelhos, 300, Boa Vista, Recife, Brazil, Postcode 500070-550.

For peer review only

João G Alves

Pediatrician

Correspondence to:

João Guilherme Alves

Instituto de Medicina Integral Professor Fernando Figueira (IMIP)

Rua dos Coelhos, 300 – Boa Vista, Recife – PE. Brazil.

Postcode 500070-550.

Email: joaoguilherme@imip.org.br

Phone: +55 (81) 999746351

For peer review only

ABSTRACT

Objective: To describe the neurodevelopment of children with congenital Zika syndrome du

ring the second year of life.

Design: Case series study.

Setting: Instituto de Medicina Integral Prof. Fernando Figueira (IMIP), Pernambuco state, Brazil.

Participants: 24 children with congenital Zika syndrome born with microcephaly during the Zika outbreak in Brazil, 2015 and followed up at IMIP during their second year of life.

Main outcome measure: Denver development test II, head circumference and clinical neurological examination.

Results: All children presented neurodevelopment delay: for an average chronological age of 19.9 months the equivalent age for language was 2.1 months, gross motor 2.7 months, fine motor/adaptative 3.1 months and personal/social 3.4 months. Head circumference maintained below the 3rd percentile for age and gender; growth rate up to the second year of life was 10.3cm (expected growth 13cm). Muscle tone was increased in 23/24 (95.5%) of children, musculo-tendinous reflexes were increased in the whole sample and clonus was present in 18/24 (77.3%). All except one had epilepsy.

Conclusion: Children born with microcephaly associated with congenital Zika vírus have a significant delay in neurodevelopment.

Key-words: Zika virus, congenital Zika syndrome, neurodevelopment

Strengths and limitations of this study

- A good-sized cohort of children with congenital Zika syndrome followed up 2 years of age;
- An important question was answered: "What developmental problems do children with congenital Zika syndrome experience 2 years post diagnosis?"

Limitation

- Children with congenital Zika syndrome but with less severe symptoms at birth were not studied.

For peer review only

Introduction

After the outbreak of congenital Zika syndrome in Brazil (2015/2016), healthcare professionals were faced with a population of children with microcephalic and many neurological manifestations (1,2). These children may present with epilepsy, abnormalities of tone or movement, including marked hypertonia and signs of extrapyramidal involvement, congenital limb contractures, dysphagia, sensorineural hearing loss and visual involvement (3, 4). However there are no published follow-up studies reporting the neurodevelopment, head growth and evolution of neurologic manifestations in children with congenital Zika syndrome. Some of these children have been followed at IMIP and they are now around two years old. Our aim is to describe the neurodevelopment, head growth and neurologic clinical manifestations of these children with congenital Zika syndrome during the first two years of life.

For peer review

Methods

A case series study was conducted at Instituto de Medicina Integral Prof. Fernando Figueira (IMIP), Recife, Brazil, between January and August 2017. IMIP is the largest hospital in northeastern Brazil. The first cases of Zika vírus outbreak in Brazil associated with microcephaly were registered in IMIP. From August 2015 to March 2016, 3,440 children were born at IMIP and 178 had microcephaly associated with congenital Zika virus infection. At the time of this study 109 children born between this period with microcephaly and congenital Zika syndrome were being followed at the Department of Paediatric Neurology of IMIP. 24 children were close to completing the second year of life and are presented in this study. These children were born with microcephaly

and congenital Zika syndrome, as defined by the WHO (https://www.cdc.gov/pregnancy/zika/research/microcephaly-case-definitions.html5). Between the age of 18 and 24 months the children underwent a complete neurological evaluation including the application of the Denver development test II. This study was approved by IMIP Research Ethical Committee (CAAE 6167876.1.0000.5201) and an informed consent was signed by the parents of the studied children.

All children were born with a head circumference below the 3rd percentile for gestational age and gender. Congenital Zika syndrome was characterized by microcephaly associated with the following neuroimages pattern: cerebral calcifications, ventriculomegaly, malformation of cortical development, hypoplasia of the cerebellum or brainstem, and abnormalities of white matter; laboratory findings excluded STORCH (syphilis, toxoplasmosis, rubella, cytomegalovirus, and herpesvirus) infections in the mother or baby, or both; and a serum or cerebrospinal fluid sample of the infant positive for IgM anti-Zika .

A clinical evaluation included a complete physical and neurological examination (history, posture, cranial nerves, muscle tone, sensory and primitive reflexes), measurement of head circumference and psychomotor development assessment by checking if the chronological acquisition of the main neurological milestones were appropriated for age. The Denver development screening test II was also applied. This test is a screening tool used routinely in pediatric care to assess developmental milestones in children ages 0-6 years old. Denver development screening test II has high inter-rater reliability and includes 125 itens in four sections: psico-social (aspects

For peer review only

regarding the socialization of the child inside and outside the family environment), language (production of sound, ability to recognize, understand and use the language), gross motor (body motor control, how to sit and walk) and fine motor adaptive (eye-hand coordination, manipulation of small objects). The possible outcomes were classified as normal, delay and non-testable. Clinical exam and Denver development screening test II were applied by a neuropediatrician specialist. To address potential sources of bias the same neuropediatriacian (LVA) did multiple neurological examination throughout the two years of the children followed.

Patient and Public Involvement

All families were previously informed about the research question and the outcome measures. Patients were not involved in developing plans for recruitment, design, or implementation of the study. The results of the research were disseminated to study participants.

Results

24 children with congenital Zika syndrome were evaluated. 14 (58.3%) of the children were female; age ranged from 16 to 24 months (19.7 m). Mothers age ranged from 15 to 39 years and 10/24 (41.6%) were primiparus; 17/24 had a prenatal diagnostic of microcephaly by ultrasound . Some mothers reported fever (9/24; 40%) and rash (13/24; 54%) during the first half of pregnancy.

For peer review only

Two children were preterm (31 and 35 gestational week). All infants were fed by gastric tube and 6/24 (25%) were malnourished.

Head circumference growth is shown at table 1; mean growth of head perimeter from birth to the age of clinical evaluation was 10.3 cm. 8/24 (33.3%) of the children had hospital admissions during their first 2 years of life: non-controlled epilepsy (2), ventriculoperitoneal shunt (2) and infection (3) – diarrhoea, urinary tract infection and pneumonia. Almost all children (23/24) had recurrent episodes of convulsion and are receiving treatment with anti-epileptic drugs.

Neurologic evaluation showed that all children presented impairment of neuropsychomotor development; none stood with support, walked or were able to say a word. Denver screening test II results according to the equivalent age are shown at table 2; greater impairment was observed in language and lower impairment in psycho-social section. The mean age of the clinical examination was 19.9 months and equivalent age for language, gross motor, fine motor/adaptative and personal/social were respectively: 2.1 months, 2.7 months, 3.1 months and 3.4 months. All children attend a weekly session of motor physiotherapy, occupational therapy and speech therapy, as well as families receiving psychological support.

Tone was increased in 23/24 (95.5%) of children, musculo-tendinous reflexes were increased in the whole sample and clonus was present in 18/24 (77.3%).

Discussion

For peer review only

A severe impairment of the neuropsychomotor development of children with congenital Zika syndrome was observed. The 24 studied children around 2 years of age could not stand alone, walk or say a word – these are expected developmental milestones for the studied age rangeIt should be noted that these children were followed up in a teaching hospital and were assisted by several health professionals including physicians, physiotherapists, speech therapists, occupational therapists, psychologists and nurses. This seems to assume that the prognosis of these children is very poor despite the intensive therapeutic support.

Congenital microcephaly regardless of cause presents a significant risk for delay across all aspects of development and for long term disability. Serious development delay was found in all 24 studied children with congenital Zika sindrome around 2 years of age. Gordon-Lipkine et al (5) studying children with congenital microcephaly of different etiologies found that 16/22 (73%) had delay in development: gross motor (65%), fine-motor (59%) and language (59%). Perhaps the fact that we had studied children with severe microcephaly explains this difference. In addition, the evolution of children with microcephaly associated with Zika virus is still unknown as this syndrome has only recently been described. Our report is a pioneer study describing the development during the first two years of life of children born with microcephaly associated with Zika virus.

All children studied had microcephaly associated with neuroimaging abnormalities, positive serology for Zika virus and other congenital infections (STORCH) were excluded. Moreover, they did not present syndromic facies or congenital malformations. All this seems to confirm the Zika virus as an

etiological agent of our case series study. Besides microcephaly the head growth during the first months of life remained below the normal values (observed 10.3 cm and expected 13/14cm) and all children kept a head circumference below the 3rd percentile for age and gender.

Zika virus infects neural progenitor cells leading to less cell migration, neurogenesis impairment, cell death and, consequently, microcephaly in newborns (6). Additionally there is some evidence that Zika virus can continue to replicate in fetal brains during the first months of extra-uterine life (7). All this seems to cause damage for the rest of life.

Almost all studied children has epilepsy. According to a recent review 54% of the children with congenital Zika infection develop epileptic seizures during the first year of life (8). Maybe this difference can be attributed to the fact that we only studied children with severe microcephaly and have followed these children over two years of age. Some studies have shown that convulsive epilepsy worsens the development delay of children with microcephaly (9).

The Denver development test II is culturally adapted to Brazil and has high inter-rater reliability (10). The Denver test II was applied in children with ages between 17 and 24 months and results obtained were equivalent to the development for the age of 1 to 8 months. The Denver II categorizes a child's performance as "Delay" (a child failing an element which ≥90% of children who are his/her age would pass) (11). Our results for the Denver test compared to other studies with other types of congenital infections (CMV and Rubella) have shown more disappointing results

For peer review only

(12, 13). This means that a serious impact on the development is associated with congenital Zika syndrome.

Our study had strength and limitations. For the first time the neurodevelopment of infants born with microcephaly associated with Zika virus is reported. Unfortunately, due to operational difficulties we were unable to study all 57 children followed at IMIP and close to completing the two years of age. It should be noted that many children used drugs to treat epilepsy and that this may interfere with responses to the Denver test. However the Denver development test II was applied by only one evaluator and the delays in the marks of neurodevelopment were very clearAdditionally, the same neuro-pediatrician did multiple neurological examination throughout the two years the children were followed. Another limitation is that we only studied children with microcephaly, constituting severe congenital Zika syndrome. It is speculated that microcephaly is likely an end-point of this devastating congenital infection. Long-term studies are needed to assess the clinical relevance of brain anomalies that are encountered and the neurodevelopmental sequelae in children with congenital Zika infection without microcephaly.

Conclusion

In the sample evaluated children born with microcephaly associated with congenital Zika virus had a significant delay in neurodevelopment during their second year of life. As this study was the first to describe the neurodevelopment of children with congenital Zika syndrome other follow-up studies are needed to confirm these findings. We urgently need to optimize the provision of healthcare and improve the quality of life of these patients.

For peer review only

Contributors

LVA and JGM contributed to conception of the work; LVA, CEP and GVS, contributed to the acquisition of data; LVA, JGM, JGA contributed to the analysis and interpretation of data for the work; and LVA is the guarantor. LVA, CEP, GVS, JGM and JGA drafted the work and revised it critically. LVA, CEP, GVS, JGM and JGA approved the version to be published.

Funding

No external funding provided.

Competing interests

All authors have completed the ICMJE uniform disclosure form at www.icmje.org/coi_disclosure.pdf and declare: no support from any organisation for the submitted work; no financial relationships with any organisations that might have an interest in the submitted work in the previous three years; no other relationships or activities that could appear to have influenced the submitted work.

Ethical approval

This study was approved by the IMIP ethics committee and the children's carers gave informed consent for the publication of the results.

Data sharing

Clinical data is available from the corresponding author at joaoguilherme@imip.org.br

Transparency

The lead author (LVA) affirms that the manuscript is an honest, accurate, and transparent account of the study being reported; that no important aspects of the study have been omitted; and that any discrepancies from the study as planned have been explained.

References

1 - Passi D, Sharma S, Dutta SR, Ahmed M. Zika Virus Diseases - The New Face of an Ancient Enemy as Global Public Health Emergency (2016): Brief Review and Recent Updates. Int J Prev Med. 2017;8:6. doi: 10.4103/2008-7802.199641.

2 – Triunfo M. Microcephaly in Brazil: confidence builds in Zika connection. Lancet Infect Dis. 2016;16(5):527-528. doi: 10.1016/S1473-3099(16)30015-9

3 - Moore CA, Staples JE, Dobyns WB, et al. Characterizing the pattern of anomalies in congenital Zika syndrome for pediatric clinicians. JAMA Pediatr 2017;171:288–95.

4 - Leal MC, Muniz LF, Ferreira TS, et al. Hearing loss in infants with microcephaly and evidence of congenital Zika virus infection—Brazil, November 2015–May 2016. MMWR Morb Mortal Wkly Rep 2016;65:917–9.*14*,*15*.

5 - Gordon-Lipkine E, Gentner MB, German R, Leppert ML. Neurodevelopmental outcomes in 22 children with microcephaly of different etiologies. J Child Neurol 2017;32(9):804-9.

6 - Saad T, PennaeCosta AA, de Góes FV, de Freitas M, de Almeida JV, de Santa Ignêz LJ, Amancio AP, Alvim RJ, Antunes Kramberger LA. Neurological manifestations of congenital Zika virus infection. Childs Nerv Syst. 2017 Nov 10. doi: 10.1007/s00381-017-3634-4.

7 - Bhatnagar J, Rabeneck DB, Martines RB, Reagan-Steiner S, Ermias Y, Estetter LB, Suzuki T, Ritter J, Keating MK, Hale G, Gary J, Muehlenbachs A, Lambert A, Lanciotti R, Oduyebo T, Meaney-Delman D, Bolaños F, Saad EA, Shieh WJ, Zaki SR. Zika Virus RNA Replication and Persistence in Brain and Placental Tissue. Emerg Infect Dis. 2017;23(3):405-414. doi: 10.3201/eid2303.161499.

8 - Pessoa A, van der Linden V, Yeargin-Allsopp M, Carvalho MDCG, Ribeiro EM, Van Naarden Braun K, Durkin MS, Pastula DM, Moore JT, Moore CA. Motor Abnormalities and Epilepsy in Infants and Children With Evidence of Congenital Zika Virus Infection. Pediatrics. 2018;141(Suppl 2):S167-S179. doi: 10.1542/peds.2017-2038F.

9 - Abdel-Salam GM, Halász AA, Czeizel AE. Association of epilepsy with different groups of microcephaly. Dev Med Child Neurol. 2000;42(11):760-7.

10 - Pinto FCA, Isotani SM, Sabatés AL, Perissinoto J. Denver II: proposed behaviors compared to those of children from São Paulo. Revista CEFAC. 2015. Vol 17(4). p.1262-1269.

11 - Frankenburg WK, Dodds J, Archer P, Shapiro H, Bresnick B. The Denver II: a major revision and restandardization of the Denver Developmental Screening Test. Pediatrics. 1992;89:91–7.

12 - Oliver SE, Cloud GA, Sánchez PJ, Demmler GJ, Dankner W, Shelton M, Jacobs RF, Vaudry W, Pass RF, Soong SJ, Whitley RJ, Kimberlin DW; National Institute of Allergy, Infectious Diseases Collaborative Antiviral Study Group. Neurodevelopmental outcomes following ganciclovir therapy in symptomatic congenital cytomegalovirus infections involving the central nervous system. J Clin Virol. 2009;46 Suppl 4:S22-6. doi: 10.1016/j.jcv.2009.08.012.

13 - Toizumi M, Nguyen GT, Motomura H, Nguyen TH, Pham E, Kaneko KI, Uematsu M, Nguyen HA, Dang DA, Hashizume M, Yoshida LM, Moriuchi H. Sensory defects and developmental delay among children with congenital rubella syndrome. Sci Rep. 2017;7:46483. doi: 10.1038/srep46483.

Tabel 1– Head circumference growth from birth to current age

Gender	Current Age (months)	Head circumference at birth (cm)	Current circumference (cm)	Head circumference expected(cm)	Head growth achieved (%)
F	19	31	41	45.7	63.6
F	18	30	38	45.6	51.2
M	21	26	36	46.4	49.0
M	20	27	39	46.3	62.5
F	21	32	41	45.8	65.2
M	22	23	37	47.5	57.1
F	20	30	37	45.8	44.3
M	19	31	44	47.2	80.2
M	17	27	35	47.0	40.0
F	20	29	43.5	45.8	86.3
F	21	28	36	45.9	44.6
M	21	29	41	46.4	68.9
F	19	26	37.5	45.7	58.3
M	19	29	45	47.2	87.9
F	19	28	37	45.7	50.8
F	19	29.5	39	45.7	58.6
M	18	27	42	47.2	74.2
F	19	31.5	42	45.7	66.9
F	22	25	35.5	47.0	47.7
F	22	31	40	47.0	56.2
M	24	29	37.5	47.7	45.4

For peer review only

F	19	30.5	42.5	45.7	78.9
M	16	30	41	45.6	70.5
F	24	31	43	47.0	75.0

For peer review only

Tabel 2– Denver Development Screening Test II in 24 children with congenital Zika syndrome

Current age (months)	Language (equivalence in age months)	Gross motor (equivalence in age months)	Fine motor/adaptative (equivalence in age months)	Personal/social (equivalence in age months)
19	1	1	1	1
18	1	4	4	4
21	1	4	4	3
20	1	1	1	3
21	4	4	4	4
22	1	1	1	3
20	1	1	1	1
19	6	7	7	6
17	1	1	1	1
20	7	7	7	6
21	1	1	1	3
21	1	4	8	8
19	1	1	4	4
19	1	1	1	3
19	4	5	4	4
19	4	1	1	4
18	1	1	1	1

For peer review only

19	1	1	1	1
22	4	4	6	6
22	3	4	4	4
24	1	4	4	3
19	1	3	3	3
16	2	2	3	3
24	2	3	3	4

For peer review only

NEURODEVELOPMENT OF 24 CHILDREN BORN IN BRAZIL WITH CONGENITAL ZIKA SYNDROME IN 2015 – CASE SERIES STUDY

Lucas V Alves, Camila E Paredes, Germanna V Silva, Júlia G Mello, João G Alves

The CARE guidelines checklist

Item name	Item no.	Brief description
Title	1	The words 'case series' appear in the title - OK
Keywords	2	The key elements of this report are included in 4 words - OK
Abstract	3	Case Presentation:<br>– The main symptoms, clinical findings, diagnoses and interventions and main outcomes - OK<br>Conclusion – OK
Introduction	4	Brief background summary of this case referencing the relevant medical literature – OK
Patient	5	• a) Demographic information (eg, age, gender) - OK

For peer review only

Item name	Item no.	Brief description
information		• b) Main symptoms of the patient - OK<br>• c) Medical, family, and psychosocial history—including diet, lifestyle, and details about relevant comorbidities including past interventions and their outcomes – OK
Clinical findings	6	Describe the relevant physical examination (PE) findings – OK
Timeline	7	Depict important dates and times in this case (table or figure) - OK
Diagnostic assessment	8	• a) Diagnostic methods (eg, PE, laboratory testing, imaging, questionnaires) - OK<br>• b) Diagnostic challenges (eg, financial, language/cultural) - OK<br>• c) Diagnostic reasoning including other diagnoses considered - OK<br>• d) Prognostic characteristics (eg, staging) where applicable - OK
Therapeutic intervention	9	• a) Types of intervention (eg, pharmacologic, surgical, preventive, self-care)<br>• – Administration of intervention (eg, dosage, strength, duration) - OK<br>• – Changes in intervention (with rationale) - OK
Follow-up and outcomes	10	• a) Summarise the clinical course of all follow-up visits including<br>• – Clinician and patient-assessed outcomes - OK<br>• – Important follow-up test results (positive or negative) - OK<br>• – Intervention adherence and tolerability (and how this was assessed) - OK<br>• – Adverse and unanticipated events - OK

For peer review only

Item name	Item no.	Brief description
Discussion	11	• a) The strengths and limitations of the management of this case - OK<br>• b) The relevant medical literature - OK<br>• c) The rationale for conclusions (including assessments of cause and effect) - OK<br>• d) The main 'take-away' lessons of this case report – OK
Patient perspective	12	The patient should share his or her perspective or experience whenever possible - OK
Informed consent	13	Did the patient give informed consent? Please provide if requested – OK

For peer review only

BMJ Open

NEURODEVELOPMENT OF 24 CHILDREN BORN IN BRAZIL WITH CONGENITAL ZIKA SYNDROME IN 2015 – CASE SERIES STUDY

Journal:	*BMJ Open*
Manuscript ID	bmjopen-2017-021304.R2
Article Type:	Research
Date Submitted by the Author:	01-Jun-2018

Complete List of Authors:	Alves, Lucas; Instituto de Medicina Integral Professor Fernando Figueira, Paediatric Neurology<br>Paredes, Camila; Faculdade Pernambucana de Saude, Teaching Department<br>Silva, Germanna; Faculdade Pernambucana de Saude<br>Mello, Julia; Instituto de Medicina Integral Professor Fernando Figueira, Department of Paediatrics<br>Alves, João; IMIP, Department of Paediatrics

**Primary Subject Heading**:	Infectious diseases
Secondary Subject Heading:	Neurology, Paediatrics
Keywords:	Developmental neurology & neurodisability < PAEDIATRICS, Paediatric infectious disease & immunisation < PAEDIATRICS, Paediatric neurology < PAEDIATRICS

SCHOLARONE™
Manuscripts

NEURODEVELOPMENT OF 24 CHILDREN BORN IN BRAZIL WITH CONGENITAL ZIKA SYNDROME IN 2015 – CASE SERIES STUDY

Lucas V Alves, Camila E Paredes, Germanna C Silva, Júlia G Mello, João G Alves

Department of Paediatrics Neurology, Instituto de Medicina Integral Prof. Fernando Figueira (IMIP), Rua dos Coelhos, 300, Boa Vista, Recife, Brazil, Postcode 50070-550 Lucas V Alves

Pediatric Neurologist

Teaching Department, Faculdade Pernambucana de Saúde (FPS) Rua Jean Émile Favre, 422,333 Imbiribeira, Recife, Brazil, Postcode 51200-060 Camila E Paredes

Medical Student

Teaching Department, Faculdade Pernambucana de Saúde (FPS), Rua Jean Émile Favre, 422, Imbiribeira, Recife, Brazil, Postcode 51200-060. Germanna V Silva

Medical Student

Department of Paediatrics, Instituto de Medicina Integral Prof. Fernando Figueira (IMIP), Rua dos Coelhos, 300, Boa Vista, Recife, Brazil, Postcode 500070-550 Júlia G Mello,

Pediatrician

Department of Pediatrics, Instituto de Medicina Integral Prof. Fernando Figueira (IMIP), Rua dos Coelhos, 300, Boa Vista, Recife, Brazil, Postcode 500070-550.

For peer review only

João G Alves

Pediatrician

Correspondence to:

João Guilherme Alves

Instituto de Medicina Integral Professor Fernando Figueira (IMIP)

Rua dos Coelhos, 300 – Boa Vista, Recife – PE. Brazil.

Postcode 500070-550.

Email: joaoguilherme@imip.org.br

Phone: +55 (81) 999746351

For peer review only

ABSTRACT

Objective: To describe the neurodevelopment of children with congenital Zika syndrome du

ring the second year of life.

Design: Case series study.

Setting: Instituto de Medicina Integral Prof. Fernando Figueira (IMIP), Pernambuco state, Brazil.

Participants: 24 children with congenital Zika syndrome born with microcephaly during the Zika outbreak in Brazil, 2015 and followed up at IMIP during their second year of life.

Main outcome measure: Denver development test II, head circumference and clinical neurological examination.

Results: All children presented neurodevelopment delay: for an average chronological age of 19.9 months the equivalent age for language was 2.1 months, gross motor 2.7 months, fine motor/adaptative 3.1 months and personal/social 3.4 months. Head circumference maintained below the 3rd percentile for age and gender; growth rate up to the second year of life was 10.3cm (expected growth 13cm). Muscle tone was increased in 23/24 (95.5%) of children, musculo-tendinous reflexes were increased in the whole sample and clonus was present in 18/24 (77.3%). All except one had epilepsy.

Conclusion: Children born with microcephaly associated with congenital Zika vírus have a significant delay in neurodevelopment.

Key-words: Zika virus, congenital Zika syndrome, neurodevelopment

Strengths and limitations of this study

- A good-sized cohort of children with congenital Zika syndrome followed up 2 years of age;
- All children were born with microcephaly and between the age of 18 and 24 months underwent a complete neurological evaluation including the application of the Denver development test II.

Limitation

- Children with congenital Zika infection but asymptomatic or with less severe symptoms at birth were not studied.

For peer review only

Introduction

After the Zika outbreak in Brazil (2015/2016), healthcare professionals were faced with a population of children with congenital Zika syndrome, born with microcephalic and many neurological manifestations (1,2). These children may present with epilepsy, abnormalities of tone or movement, including marked hypertonia and signs of extrapyramidal involvement, congenital limb contractures, dysphagia, sensorineural hearing loss and visual involvement (3, 4). However there are no published follow-up studies reporting the neurodevelopment, head growth and evolution of neurologic manifestations in children with congenital Zika syndrome. Some of these children have been followed at IMIP and they are now around two years old. Our aim is to describe the neurodevelopment, head growth and neurologic clinical manifestations of these children with congenital Zika syndrome during the first two years of life.

For peer review

Methods

A case series study was conducted at Instituto de Medicina Integral Prof. Fernado Figueira (IMIP), Recife, Brazil, between January and August 2017. IMIP is the largest hospital in northeastern Brazil. The first cases of Zika vírus outbreak in Brazil associated with microcephaly were registered in IMIP. From August 2015 to March 2016, 3,440 children were born at IMIP and 178 had microcephaly associated with congenital Zika virus infection. At the time of this study 109 children born between this period with microcephaly and congenital Zika syndrome were being followed at the Department of Paediatric Neurology of IMIP. 24 children were close to completing the second year of life and are presented in this study. These children were born with microcephaly

and congenital Zika syndrome, as defined by the WHO (https://www.cdc.gov/pregnancy/zika/research/microcephaly-case-definitions.html5). Between the age of 18 and 24 months the children underwent a complete neurological evaluation including the application of the Denver development test II. This study was approved by IMIP Research Ethical Committee (CAAE 6167876.1.0000.5201) and an informed consent was signed by the parents of the studied children.

All children were born with a head circumference below the 3rd percentile for gestational age and gender. Congenital Zika syndrome was characterized by microcephaly associated with the following neuroimages pattern: cerebral calcifications, ventriculomegaly, malformation of cortical development, hypoplasia of the cerebellum or brainstem, and abnormalities of white matter; laboratory findings excluded STORCH (syphilis, toxoplasmosis, rubella, cytomegalovirus, and herpesvirus) infections in the mother or baby, or both; and a serum or cerebrospinal fluid sample of the infant positive for IgM anti-Zika .

A clinical evaluation included a complete physical and neurological examination (history, posture, cranial nerves, muscle tone, sensory and primitive reflexes), measurement of head circumference and psychomotor development assessment by checking if the chronological acquisition of the main neurological milestones were appropriated for age. The Denver development screening test II was also applied. This test is a screening tool used routinely in pediatric care to assess developmental milestones in children ages 0-6 years old. Denver development screening test II has high inter-rater reliability and includes 125 itens in four sections: psico-social (aspects

For peer review only

regarding the socialization of the child inside and outside the family environment), language (production of sound, ability to recognize, understand and use the language), gross motor (body motor control, how to sit and walk) and fine motor adaptive (eye-hand coordination, manipulation of small objects). The possible outcomes were classified as normal, delay and non-testable. Clinical exam and Denver development screening test II were applied by a neuropediatrician specialist. To address potential sources of bias the same neuropediatriacian (LVA) did multiple neurological examination throughout the two years of the children followed.

Patient and Public Involvement

All families were previously informed about the research question and the outcome measures. Patients were not involved in developing plans for recruitment, design, or implementation of the study. The results of the research were disseminated to study participants.

Results

24 children with congenital Zika syndrome were evaluated. 14 (58.3%) of the children were female; age ranged from 16 to 24 months (19.7 m). Mothers age ranged from 15 to 39 years and 10/24 (41.6%) were primiparus; 17/24 had a prenatal diagnostic of microcephaly by ultrasound . Some mothers reported fever (9/24; 40%) and rash (13/24; 54%) during the first half of pregnancy.

For peer review only

Two children were preterm (31 and 35 gestational week). All infants were fed by gastric tube and 6/24 (25%) were malnourished.

Head circumference growth is shown at table 1; mean growth of head perimeter from birth to the age of clinical evaluation was 10.3 cm. 8/24 (33.3%) of the children had hospital admissions during their first 2 years of life: non-controlled epilepsy (2), ventriculoperitoneal shunt (2) and infection (3) – diarrhoea, urinary tract infection and pneumonia. Almost all children (23/24) had recurrent episodes of convulsion and are receiving treatment with anti-epileptic drugs.

Neurologic evaluation showed that all children presented impairment of neuropsychomotor development; none stood with support, walked or were able to say a word. Denver screening test II results according to the equivalent age are shown at table 2; greater impairment was observed in language and lower impairment in psycho-social section. The mean age of the clinical examination was 19.9 months and equivalent age for language, gross motor, fine motor/adaptative and personal/social were respectively: 2.1 months, 2.7 months, 3.1 months and 3.4 months. All children attend a weekly session of motor physiotherapy, occupational therapy and speech therapy, as well as families receiving psychological support.

Tone was increased in 23/24 (95.5%) of children, musculo-tendinous reflexes were increased in the whole sample and clonus was present in 18/24 (77.3%).

Discussion

For peer review only

A severe impairment of the neuropsychomotor development of children with congenital Zika syndrome was observed. The 24 studied children around 2 years of age could not stand alone, walk or say a word – these are expected developmental milestones for the studied age rangeIt should be noted that these children were followed up in a teaching hospital and were assisted by several health professionals including physicians, physiotherapists, speech therapists, occupational therapists, psychologists and nurses. This seems to assume that the prognosis of these children is very poor despite the intensive therapeutic support.

Congenital microcephaly regardless of cause presents a significant risk for delay across all aspects of development and for long term disability. Serious development delay was found in all 24 studied children with congenital Zika sindrome around 2 years of age. Gordon-Lipkine et al (5) studying children with congenital microcephaly of different etiologies found that 16/22 (73%) had delay in development: gross motor (65%), fine-motor (59%) and language (59%). Perhaps the fact that we had studied children with severe microcephaly explains this difference. In addition, the evolution of children with microcephaly associated with Zika virus is still unknown as this syndrome has only recently been described. Our report is a pioneer study describing the development during the first two years of life of children born with microcephaly associated with Zika virus.

All children studied had microcephaly associated with neuroimaging abnormalities, positive serology for Zika virus and other congenital infections (STORCH) were excluded. Moreover, they did not present syndromic facies or congenital malformations. All this seems to confirm the Zika virus as an

etiological agent of our case series study. Besides microcephaly the head growth during the first months of life remained below the normal values (observed 10.3 cm and expected 13/14cm) and all children kept a head circumference below the 3rd percentile for age and gender.

Zika virus infects neural progenitor cells leading to less cell migration, neurogenesis impairment, cell death and, consequently, microcephaly in newborns (6). Additionally there is some evidence that Zika virus can continue to replicate in fetal brains during the first months of extra-uterine life (7). All this seems to cause damage for the rest of life.

Almost all studied children has epilepsy. According to a recent review 54% of the children with congenital Zika infection develop epileptic seizures during the first year of life (8). Maybe this difference can be attributed to the fact that we only studied children with severe microcephaly and have followed these children over two years of age. Some studies have shown that convulsive epilepsy worsens the development delay of children with microcephaly (9).

The Denver development test II is culturally adapted to Brazil and has high inter-rater reliability (10). The Denver test II was applied in children with ages between 17 and 24 months and results obtained were equivalent to the development for the age of 1 to 8 months. The Denver II categorizes a child's performance as "Delay" (a child failing an element which ≥90% of children who are his/her age would pass) (11).

Our results for the Denver test compared to other studies with other types of congenital infections (CMV and Rubella) have shown more disappointing results

For peer review only

(12, 13). This means that a serious impact on the development is associated with congenital Zika syndrome.

Our study had strength and limitations. For the first time the neurodevelopment of infants born with microcephaly associated with Zika virus is reported. Unfortunately, due to operational difficulties we were unable to study all 57 children followed at IMIP and close to completing the two years of age. It should be noted that many children used drugs to treat epilepsy and that this may interfere with responses to the Denver test. However the Denver development test II was applied by only one evaluator and the delays in the marks of neurodevelopment were very clearAdditionally, the same neuro-pediatrician did multiple neurological examination throughout the two years the children were followed. Another limitation is that we only studied children with microcephaly, constituting severe congenital Zika syndrome. It is speculated that microcephaly is likely an end-point of this devastating congenital infection. Long-term studies are needed to assess the clinical relevance of brain anomalies that are encountered and the neurodevelopmental sequelae in children with congenital Zika infection without microcephaly.

Conclusion

In the sample evaluated children born with microcephaly associated with congenital Zika virus had a significant delay in neurodevelopment during their second year of life. As this study was the first to describe the neurodevelopment of children with congenital Zika syndrome other follow-up studies are needed to confirm these findings. We urgently need to optimize the provision of healthcare and improve the quality of life of these patients.

For peer review only - http://bmjopen.bmj.com/site/about/guidelines.xhtml

Contributors

LVA and JGM contributed to conception of the work; LVA, CEP and GCS, contributed to the acquisition of data; LVA, JGM, JGA contributed to the analysis and interpretation of data for the work; and LVA is the guarantor. LVA, CEP, GCS, JGM and JGA drafted the work and revised it critically. LVA, CEP, GCS, JGM and JGA approved the version to be published.

Funding

No external funding provided.

Competing interests

All authors have completed the ICMJE uniform disclosure form at www.icmje.org/coi_disclosure.pdf and declare: no support from any organisation for the submitted work; no financial relationships with any organisations that might have an interest in the submitted work in the previous three years; no other relationships or activities that could appear to have influenced the submitted work.

Ethical approval

This study was approved by the IMIP ethics committee and the children's carers gave informed consent for the publication of the results.

Data sharing statement

Clinical data is available from the corresponding author at joaoguilherme@imip.org.br

ransparency

The lead author (LVA) affirms that the manuscript is an honest, accurate, and transparent account of the study being reported; that no important aspects of the study have been omitted; and that any discrepancies from the study as planned have been explained.

References

1 - Passi D, Sharma S, Dutta SR, Ahmed M. Zika Virus Diseases - The New Face of an Ancient Enemy as Global Public Health Emergency (2016): Brief Review and Recent Updates. Int J Prev Med. 2017;8:6. doi: 10.4103/2008-7802.199641.

2 – Triunfo M. Microcephaly in Brazil: confidence builds in Zika connection. Lancet Infect Dis. 2016;16(5):527-528. doi: 10.1016/S1473-3099(16)30015-9

3 - Moore CA, Staples JE, Dobyns WB, et al. Characterizing the pattern of anomalies in congenital Zika syndrome for pediatric clinicians. JAMA Pediatr 2017;171:288–95.

4 - Leal MC, Muniz LF, Ferreira TS, et al. Hearing loss in infants with microcephaly and evidence of congenital Zika virus infection—Brazil, November 2015–May 2016. MMWR Morb Mortal Wkly Rep 2016;65:917–9.*14*,*15*.

5 - Gordon-Lipkine E, Gentner MB, German R, Leppert ML. Neurodevelopmental outcomes in 22 children with microcephaly of different etiologies. J Child Neurol 2017;32(9):804-9.

6 - Saad T, PennaeCosta AA, de Góes FV, de Freitas M, de Almeida JV, de Santa Ignêz LJ, Amancio AP, Alvim RJ, Antunes Kramberger LA. Neurological manifestations of congenital Zika virus infection. Childs Nerv Syst. 2017 Nov 10. doi: 10.1007/s00381-017-3634-4.

7 - Bhatnagar J, Rabeneck DB, Martines RB, Reagan-Steiner S, Ermias Y, Estetter LB, Suzuki T, Ritter J, Keating MK, Hale G, Gary J, Muehlenbachs A, Lambert A, Lanciotti R, Oduyebo T, Meaney-Delman D, Bolaños F, Saad EA, Shieh WJ, Zaki SR. Zika Virus RNA Replication and Persistence in Brain and Placental Tissue. Emerg Infect Dis. 2017;23(3):405-414. doi: 10.3201/eid2303.161499.

8 - Pessoa A, van der Linden V, Yeargin-Allsopp M, Carvalho MDCG, Ribeiro EM, Van Naarden Braun K, Durkin MS, Pastula DM, Moore JT, Moore CA. Motor Abnormalities and Epilepsy in Infants and Children With Evidence of Congenital Zika Virus Infection. Pediatrics. 2018;141(Suppl 2):S167-S179. doi: 10.1542/peds.2017-2038F.

9 - Abdel-Salam GM, Halász AA, Czeizel AE. Association of epilepsy with different groups of microcephaly. Dev Med Child Neurol. 2000;42(11):760-7.

10 - Pinto FCA, Isotani SM, Sabatés AL, Perissinoto J. Denver II: proposed behaviors compared to those of children from São Paulo. Revista CEFAC. 2015. Vol 17(4). p.1262-1269.

11 - Frankenburg WK, Dodds J, Archer P, Shapiro H, Bresnick B. The Denver II: a major revision and restandardization of the Denver Developmental Screening Test. Pediatrics. 1992;89:91–7.

12 - Oliver SE, Cloud GA, Sánchez PJ, Demmler GJ, Dankner W, Shelton M, Jacobs RF, Vaudry W, Pass RF, Soong SJ, Whitley RJ, Kimberlin DW; National Institute of Allergy, Infectious Diseases Collaborative Antiviral Study Group. Neurodevelopmental outcomes following ganciclovir therapy in symptomatic congenital cytomegalovirus infections involving the central nervous system. J Clin Virol. 2009;46 Suppl 4:S22-6. doi: 10.1016/j.jcv.2009.08.012.

13 - Toizumi M, Nguyen GT, Motomura H, Nguyen TH, Pham E, Kaneko KI, Uematsu M, Nguyen HA, Dang DA, Hashizume M, Yoshida LM, Moriuchi H. Sensory defects and developmental delay among children with congenital rubella syndrome. Sci Rep. 2017;7:46483. doi: 10.1038/srep46483.

Tabel 1– Head circumference growth from birth to current age

Gender	Current Age (months)	Head circumference at birth (cm)	Current circumference (cm)	Head circumference expected(cm)	Head growth achieved (%)
F	19	31	41	45.7	63.6
F	18	30	38	45.6	51.2
M	21	26	36	46.4	49.0
M	20	27	39	46.3	62.5
F	21	32	41	45.8	65.2
M	22	23	37	47.5	57.1
F	20	30	37	45.8	44.3
M	19	31	44	47.2	80.2
M	17	27	35	47.0	40.0
F	20	29	43.5	45.8	86.3
F	21	28	36	45.9	44.6
M	21	29	41	46.4	68.9
F	19	26	37.5	45.7	58.3
M	19	29	45	47.2	87.9
F	19	28	37	45.7	50.8
F	19	29.5	39	45.7	58.6
M	18	27	42	47.2	74.2

For peer review only

F	19	31.5	42	45.7	66.9
F	22	25	35.5	47.0	47.7
F	22	31	40	47.0	56.2
M	24	29	37.5	47.7	45.4
F	19	30.5	42.5	45.7	78.9
M	16	30	41	45.6	70.5
F	24	31	43	47.0	75.0

For peer review only

Tabel 2– Denver Development Screening Test II in 24 children with congenital Zika syndrome

Current age (months)	Language (equivalence in age months)	Gross motor (equivalence in age months)	Fine motor/adaptative (equivalence in age months)	Personal/social (equivalence in age months)
19	1	1	1	1
18	1	4	4	4
21	1	4	4	3
20	1	1	1	3
21	4	4	4	4
22	1	1	1	3
20	1	1	1	1
19	6	7	7	6
17	1	1	1	1
20	7	7	7	6
21	1	1	1	3
21	1	4	8	8
19	1	1	4	4
19	1	1	1	3
19	4	5	4	4
19	4	1	1	4
18	1	1	1	1

For peer review only

19	1	1	1	1
22	4	4	6	6
22	3	4	4	4
24	1	4	4	3
19	1	3	3	3
16	2	2	3	3
24	2	3	3	4

For peer review only

NEURODEVELOPMENT OF 24 CHILDREN BORN IN BRAZIL WITH CONGENITAL ZIKA SYNDROME IN 2015 – CASE SERIES STUDY

Lucas V Alves, Camila E Paredes, Germanna V Silva, Júlia G Mello, João G Alves

For peer review only

The CARE guidelines checklist

Item name	Item no.	Brief description	Page
Title	1	The words ‘case series’ appear in the title – OK	1
Keywords	2	The key elements of this report are included in 4 words – OK	3
Abstract	3	Case Presentation: – The main symptoms, clinical findings, diagnoses and interventions and main outcomes – OK	3
		Conclusion – OK	3
Introduction	4	Brief background summary of this case referencing the relevant medical literature – OK	5
Patient	5	• a) Demographic information (eg, age, gender) – OK	7

Item name	Item no.	Brief description	Page
information		• b) Main symptoms of the patient – OK	7
		• c) Medical, family, and psychosocial history—including diet, lifestyle, and details about relevant comorbidities including past interventions and their outcomes – OK	8
Clinical findings	6	Describe the relevant physical examination (PE) findings – OK	8
Timeline	7	Depict important dates and times in this case (table or figure)	
Diagnostic assessment	8	• a) Diagnostic methods (eg, PE, laboratory testing, imaging, questionnaires) – OK	6
		• b) Diagnostic challenges (eg, financial, language/cultural) –OK	7
		• c) Diagnostic reasoning including other diagnoses considered –	
		• OK	7
		• d) Prognostic characteristics (eg, staging) where applicable – OK	NA
Therapeutic intervention	9	• a) Types of intervention (eg, pharmacologic, surgical, preventive, self-care) NA	
		• – Administration of intervention (eg, dosage, strength, duration) NA	
		• – Changes in intervention (with rationale) NA	
Follow-up and outcomes	10	• a) Summarise the clinical course of all follow-up visits including	
		• – Clinician and patient-assessed outcomes – OK	7
		• – Important follow-up test results (positive or negative) – OK	8
		• – Intervention adherence and tolerability (and how this was assessed) – OK	NA

Item name	Item no.	Brief description	Page
		• – Adverse and unanticipated events – OK	NA
Discussion	11	• a) The strengths and limitations of the management of this case – OK	4 and 9
		• b) The relevant medical literature – OK	9
		• c) The rationale for conclusions (including assessments of cause and effect) – OK	10
		• d) The main 'take-away' lessons of this case report – OK	10-11
Patient perspective	12	The patient should share his or her perspective or experience whenever possible – OK	8
Informed consent	13	Did the patient give informed consent? Please provide if requested – OK	12

For peer review only
